# Synthetic MFG MVIRI Level 1.5 VIS channel data of Europe from 2006–2020 for long-term climatological research

**DOI:** 10.1038/s41597-025-05578-5

**Published:** 2025-08-04

**Authors:** Ivo Jung, Sheetabh Gaurav, Jörg Bendix

**Affiliations:** Philipps Universität Marburg, Department of Geography, Marburg, 35032 Marburg, Germany

**Keywords:** Climate sciences, Environmental sciences

## Abstract

Geostationary satellites observe the Earth, providing essential data for climate research and weather forecasting. Understanding long-term changes in cloud cover is particularly important, as changes in cloud albedo can affect global temperatures directly. The Meteosat programme has been monitoring Europe and Africa since 1977, providing a good basis for long-term climatological research. Due to their different sensor characteristics, data from different satellites must be harmonized to obtain a consistent time series for long-term research. This study harmonizes the Meteosat First Generation (MFG) broadband solar channel and the two Meteosat Second Generation (MSG) solar channels using a Random Forest model to generate a long-term time series of the MFG solar channel over Central Europe from 2006 to 2020, which can be used to extend the existing MFG MVIRI VIS image time series. The RF model predicts the MVIRI solar channel well (R^2^ = 0.93). In complex terrain inaccuracies in predictions may occur. The synthesized MVIRI solar channel time series has no severe discontinuities and is available for long-term research.

## Background & Summary

The World Meteorological Organization (WMO) has confirmed that 2024 was the first year in which the global average temperature surpassed the 1.5°C threshold above preindustrial levels. Recent studies have shown that decreasing low-level cloud cover, and hence decreasing planetary albedo, can be directly linked to rising global temperatures^1^. The availability of long-term, spatially extensive data that can be used to research and analyze cloud evolution over large spatial-temporal scales is therefore of great importance. Satellite systems provide data on large spatial and temporal scales and are an essential tool for climate and environmental research to better understand the Earth-atmosphere system and the effects of climate change. They monitor the Earth’s surface and atmosphere and can be used for numerous applications such as weather forecasting, measuring atmospheric constituents, obtaining cloud or aerosol properties, detecting wildfires, and many more^2–4^. Due to changes in satellite generations and instruments with evolving characteristics, long-term satellite data are often not homogeneous. However, a harmonized time series would be useful for long-term climate research, e.g. to detect long-term climate trends. Therefore, it is important to normalize the variability between the satellite instruments from different generations of satellites to obtain a homogeneous data set. This is often referred to as “harmonization” and proves to be a difficult task^5^. The term harmonization describes an inter-calibration of data from different instruments to obtain a homogeneous, synthetic data set from one instrument^6^.

The Meteosat series of the European Space Agency (ESA) and the European Organization for the Exploitation of Meteorological Satellites (EUMETSAT) consists of geostationary satellites operating over Europe and Africa since 1977^7^. The long lifetime of the Meteosat series makes it suitable for long-term climatological studies over a period of more than 30 years. However, due to the different sensor characteristics of the Meteosat First Generation (MFG) Meteosat Visible Infra-Red Imager (MVIRI) instrument and the Meteosat Second Generation (MSG) Spinning Enhanced Visible and Infrared Imager (SEVIRI) instrument and the individual sensor degradation, inhomogeneities in the time series should be considered and corrected in advance^8,9^. The recent release of EUMETSAT’s Fundamental Climate Data Record (FCDR) includes the recalibration of Level 1.5 data, including water vapor (WV), thermal infrared (IR), and visible (VIS) spectral channels of the MVIRI instrument from July 1981 to July 2006^8,10–12^. Between the Meteosat First Generation (MFG) MVIRI FCDR Release 1 and the Meteosat Second Generation (MSG) SEVIRI data, which is operational since January 2004 until now, this extends the availability of high quality data from EUMETSAT’s geostationary weather satellite series to more than 30 years^10^.

Although the FCDR release has improved the quality of the historical MFG MVIRI data, there are still differences with the modern MSG SEVIRI data in terms of bandwidth and number of spectral channels. Recent studies have shown that it is possible to simulate MVIRI VIS broadband channel radiance using well-correlated narrowband spectral channels from other satellites within the same bandwidth^13–15^. It was shown by^15^ that a combination of MSG SEVIRI 0.6 *μ*m and 0.8 *μ*m channel radiances can be used to recreate MFG MVIRI broadband radiances. Using this framework,^16^ extended the Application Facility on Climate Monitoring (CM SAF) surface radiation climate data record (CDR) by 5 years to 2010 by using the MSG SEVIRI VIS channels to simulate MFG MVIRI broadband radiances. Similar methods were used to generate the EUMETSAT CM SAF Cloud Fractional Cover CDR (COMET), which covers the period from 1991 to 2015. For the COMET CDR, the MFG MVIRI VIS broadband channel is simulated using a linear combination of the MSG narrow-band 0.6 *μ*m and 0.8 *μ*m reflectances. In contrast to previous studies^6^ used a novel machine learning (ML) method with promising accuracy to harmonize the MFG, MVIRI, and MSG SEVIRI IR and WV channels in order to extend the MFG availability by the MSG timeline.

In this study, a Random Forest Regression (RF) machine learning approach was used to harmonize the MFG MVIRI and MSG SEVIRI VIS channels to produce a consistent, long-term MFG VIS channel time series over Central Europe with high spatiotemporal resolution. More specifically, this study has two aims: First, it aims to use the two narrow-band MSG SEVIRI VIS channels (0.6 *μ*m,  0.8 *μ*m) to predict and synthesize the broadband MFG MVIRI VIS channel (0.7 *μ*m) and provide a harmonized synthetic time series dataset covering the timeline of the MSG mission up to 2020. The second goal is to provide a robust, easy-to-use framework that can be adopted to future satellite generations like the Meteosat Third Generation (MTG). To inter-calibrate the MFG MVIRI and MSG SEVIRI VIS channels, an RF is trained using MFG and MSG VIS data from January 2004 to July 2006, where overlapping data from both satellite systems are available. The synthetic MFG MVIRI VIS image data are validated against the original MFG MVIRI VIS image data from the overlap period. The synthetic MFG MVIRI VIS image data set is freely available at the data_UMR Research repository^17^. The first section of the article explains the fundamental data, necessary processing steps, and main methods in detail. The Data Records section discusses the usability and limitations of the published data. The following Technical Validation section addresses the validation of the data set.

## Methods

**Fig. 1:**
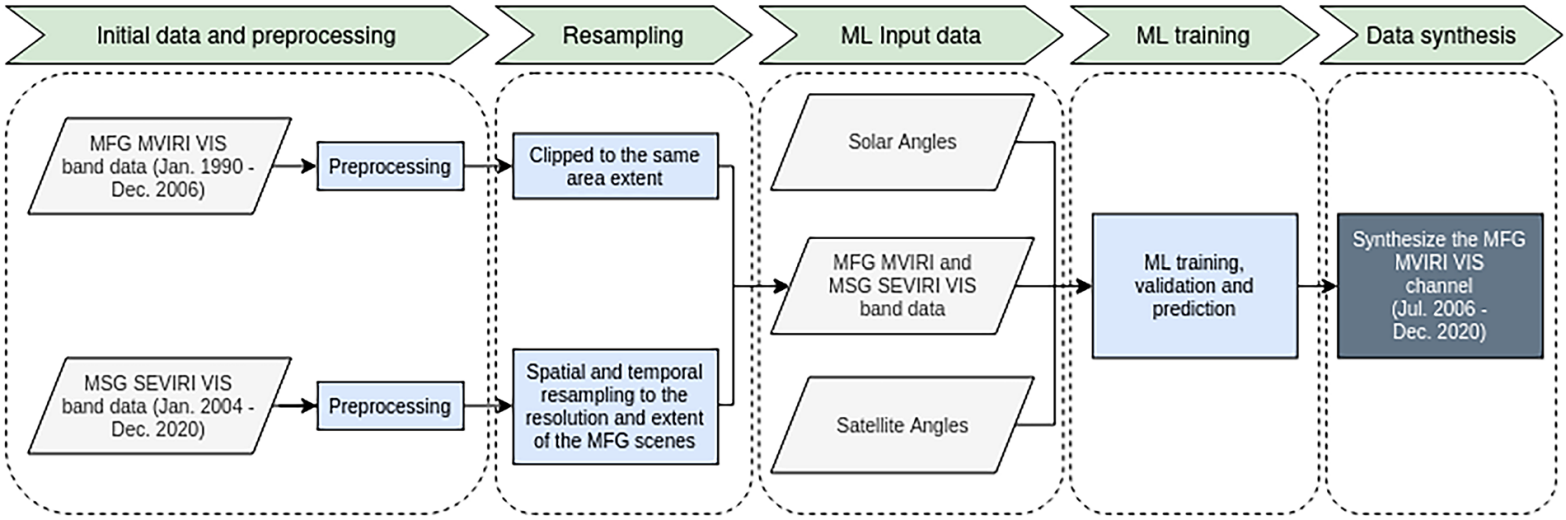
Flowchart depicting the work flow for generating the synthetic MFG MVIRI VIS band data.

Multiple steps are necessary to harmonize the MSG SEVIRI and MFG MVIRI VIS channels and create the synthetic MFG MVIRI VIS channel dataset. The general workflow is presented in Fig. 1. After the initial preprocessing steps, the MFG MVIRI and MSG SEVIRI data must be resampled to match the temporal and spatial resolutions. The data also need to be clipped to the same area extent. Next, relevant machine learning (ML) input features are calculated. These features are used alongside the VIS channel data to train an RF regression model. The model’s predictive capability and accuracy are validated. Finally, the validated model is used to synthesize the MFG MVIRI broadband VIS channel, extending its availability to the MSG timeline from July 2006 to December 2020. The data and methods are described in detail in the following subsections.

### Data and Preprocessing

**Fig. 2:**
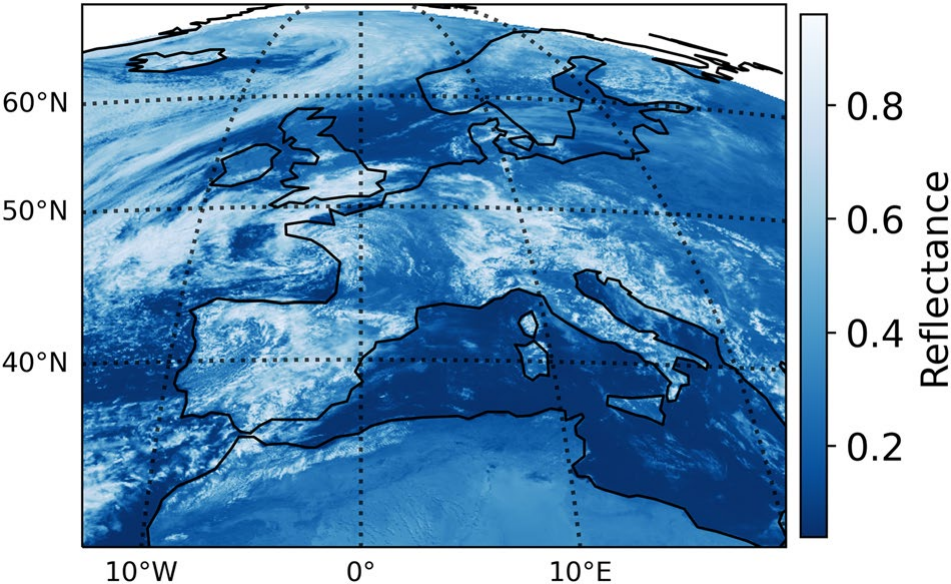
WMO Region VI (Source: WorldClim DEM^34^) with an example MFG VIS scene from June 1st 2004, 12:00 - 12:30.

This study uses the MFG MVIRI Level 1.5 FCDR^8,10–12,18^ and high-rate MSG SEVIRI Level 1.5 image data as input variables for a machine learning model to harmonize their VIS channels and generate a consistent, harmonized, synthetic MFG MVIRI Level 1.5 dataset. The datasets are open source and can be accessed and downloaded from the EUMETSAT website^18,19^. The MVIRI and SEVIRI data were calibrated, clipped to the area of central Europe (World Meteorological Organization WMO Region VI; Fig. 2), and converted to a common data format. The preprocessing was done using SatPy, a Python-based library designed to process data from Earth observation satellites^20^. Only the VIS spectral channels of the two satellite generations were used for this study. The MSG SEVIRI High Resolution Visible (HRV) channel was not used due to its significantly different spatial resolution.

The Level 1.5 MFG MVIRI image data used in this study covers a time period from January 1990 to July 2006. The VIS channel data is clipped to regions with a valid solar zenith angle (SZA)  < 90° for each pixel. In addition, pixels flagged as bad quality by an included quality flag mask are set to zero. Each satellite scene is provided in the Network Common Data Form file format (NetCDF).

**Fig. 3:**
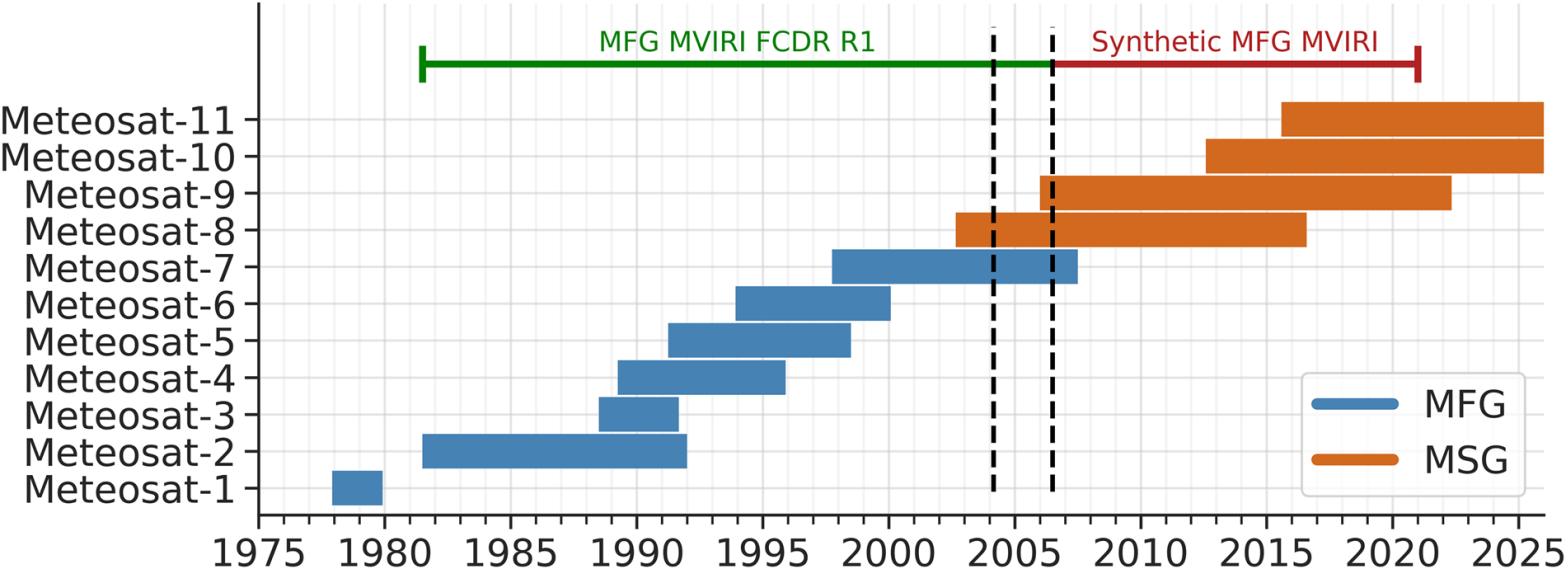
Timeline of the different EUMETSAT Meteosat satellites from the first (MFG) an second (MSG) generation. The overlapping period is highlighted between the dashed lines.

The Level 1.5 MSG SEVIRI data cover the period from January 2004 to December 2020. The files, originally in different file formats (Hierarchical Data Format HDF, Network Common Data Form NetCDF and High Rate Information Transmission HRIT), have been converted to a common file format (NetCDF). Additionally, the MSG SEVIRI data are calibrated using the calibration coefficients provided with the Level 1.5 data. The overlapping period between the MFG and MSG satellites, which was used to train the RF model, is highlighted in Fig. 3 and ranges from January 2004 to July 2006.

### Spatial and Temporal Resampling

**Fig. 4:**
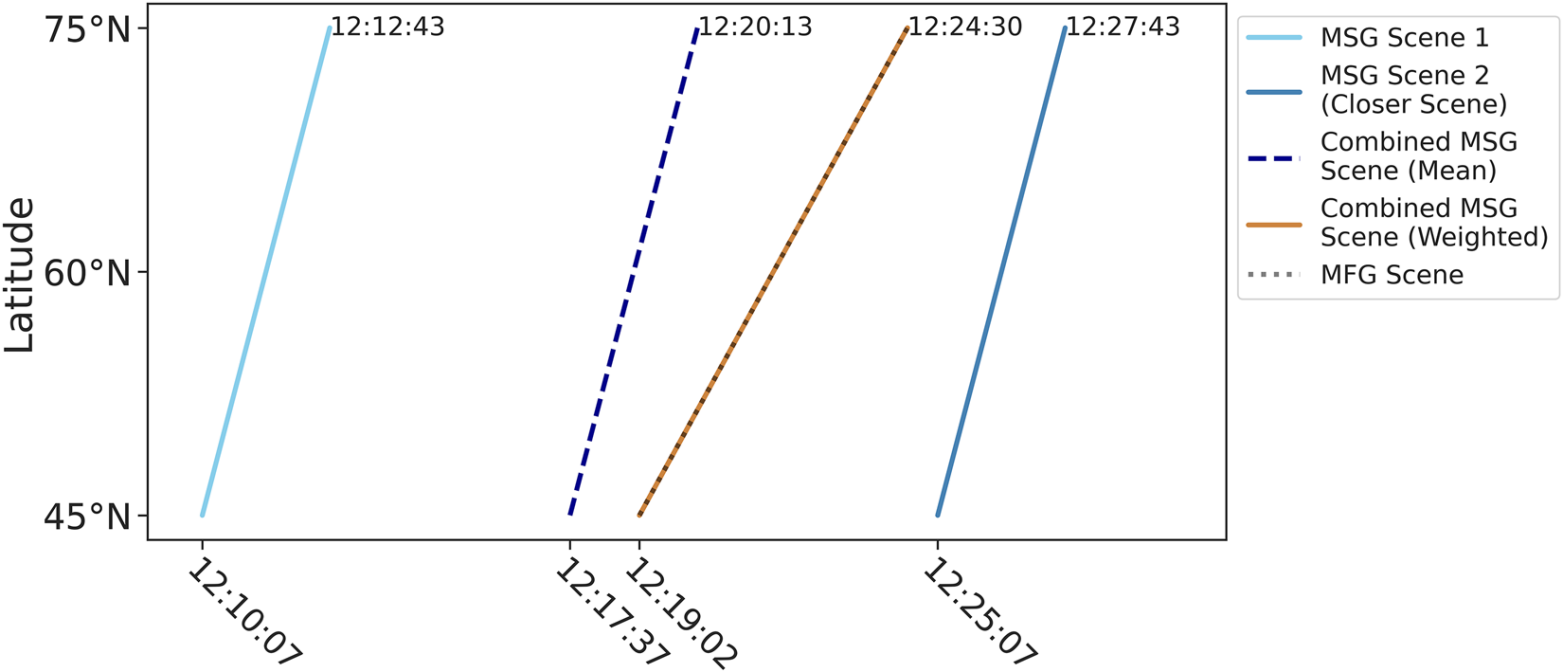
Scan time differences between MFG MVIRI and MSG SEVIRI for example scenes on June 1, 2004. Dashed lines show combined MSG scene scan times using the mean (blue) and weighted averaging that accounts for scan time differences (orange).

Due to the different temporal and spatial resolutions, the MSG and MFG VIS band data had to be temporally and spatially resampled. The MFG MVIRI VIS channel has a sampling distance at the sub-satellite point of 2.5 km and is provided as 5000 x 5000 pixel images. The MSG SEVIRI VIS channels, on the other hand, are provided as 3712  × 3712 pixel images and have a sampling distance of 3 km at nadir. Nearest Neighbor interpolation was used to match the MSG SEVIRI VIS channel resolution to the MFG MVIRI VIS channel resolution. The MSG and MFG scenes were then clipped to the same matching area extent. For the temporal resampling, the different scan times of the MFG MVIRI and MSG SEVIRI instruments have to be considered (Fig. 4). The scan time of the MVIRI instrument typically takes 5 to 6 minutes for the area of central Europe and makes a full disk scan every 30 minutes. The SEVIRI instrument onboard the MSG satellites has a shorter scan time with 2 to 3 minutes for the same area and scans the full disk every 15 minutes. Thus, there are two MSG images available for each MFG image. Furthermore there is a time shift between the two systems regarding the starting time of the scans and a difference in scanning intervals. For the temporal resampling process, the two corresponding MSG scenes every 30 minutes were linearly combined with respect to their scantime differences to the corresponding MFG scene using the following steps adopted from^6^. Step 1: Create a reference timestamp from the center of the MFG scene and calculate the scan time differences for each pixel in the scene with the reference timestamp *Δt*_*mfg*_; Step 2: Calculate the scan time differences to the MFG reference timestamp of the two MSG scenes for each pixel in the scenes *Δt*_*msg*1_ and *Δt*_*msg*2_; Step 3: Spatially resample *Δt*_*msg*1_ *Δt*_*msg*2_ to the MFG resolution using nearest neighbor interpolation; Step 4: Calculate the weighting ratios *r*1 and *r*2 for each pixel with latitude *x* and longitude *y* (Equation (1), (2)); Step 5: Combine the two MSG scenes *MSG*_1_ and *MSG*_2_ into one MSG scene *MSG*_*combined*_ using the calculated ratios *r*1 and *r*2 (Equation (3)).

1 $${r}{1}=1-\frac{(\Delta {t}_{mfg}(y,x)-\Delta {t}_{msg1}(y,x))}{\Delta {t}_{msg2}(y,x)-\Delta {t}_{msg1}(y,x)}$$

2 $${r}{2}=1-\frac{(\Delta {t}_{msg2}(y,x)-\Delta {t}_{mfg}(y,x))}{\Delta {t}_{msg2}(y,x)-\Delta {t}_{msg1}(y,x)}$$

3 $$MS{G}_{combined}=(MS{G}_{1}(y,x)\cdot r1(y,x))+(MS{G}_{2}(y,x)\cdot r2(y,x))$$

### Noise and Outliers

The original MFG and MSG data contain outliers, which are mainly located at higher latitudes above 60°N along the edges of the geostationary imaging area. They are typically caused by high off-nadir (ONA) satellite angles due to the curvature of the Earth relative to the viewing angles of the geostationary 0-degree satellites and the overall long atmospheric path and pixel distortion^21^. Therefore all pixels above the latitude of 62°N in each MFG and MSG scene were masked and removed from the data.

In addition to outliers in polar regions, low sun elevations during twilight periods (dusk or dawn) can cause noise and outliers in some areas of an image due to low angle scattering of light and poor illumination^22^. To further reduce outliers and noise in the data used for the RF model training, a daylight mask is needed to exclude twilight pixels. The daylight mask was created using a Solar Zenith Angle (SZA) threshold that excludes all pixels from each image with SZA > 80°. It was chosen based on SZA-based day-night classifications from various Meteosat products, such as the volcanic ash product which relies on the VIS channels, and other studies based on Meteosat imagery^23–25^.

### Random Forest Regression model

An RF was trained and used to synthesize the MFG VIS channel from 2004 to 2020 based on the MSG SEVIRI VIS image data. RF is a well proven and effective ML technique that can handle large data sets. It is an ensemble learning method, that uses decision trees alongside bootstrapping and aggregation. Each decision tree is build from a random subset of the training data, which ensures diversity among the decision trees. The RF algorithm aggregates the solution of individual decision trees and averages the solution of all decision trees, providing a prediction of the target variable with minimum error^26–28^. The task of training individual decision trees can be parallelized, making the algorithm efficient and applicable to large amounts of data. RF also directly provides feature importance by analyzing the impact of each input feature on the model performance, helping to select only relevant features. Additional, different tuning parameters can be adjusted independently to optimize the model performance. The RF was implemented using the well-documented, open source Python library Scikit-learn that provides state-of-the-art tools for machine learning and statistical modeling^29^. Therefore, RF was the machine learning (ML) approach of choice to harmonize the MFG and MSG VIS channels over more complex, computationally expensive models, such as neural networks. RF is easy to use, computationally efficient, and provides better results and customization than simpler methods, such as multiple linear regression models.

#### Model Input Data

The final set of RF predictors consists of different satellite viewing angles, solar angles and the MSG006 (0.6 *μ*m) and MSG008 VIS (0.8 *μ*m) channel reflectance data (Table 1). The satellite and solar angles were added because they can have effect on observed radiances due to differing atmospheric paths and scattering of light and to reduce geographic trends in the RF predictions^6,30^. The satellite viewing angles and solar angles were calculated using the Python Library SatPy^20^. The ML training, validation and test data splits were generated from 5000 random MFG and MSG scenes from the overlap period January 2004 to July 2006. After spatial and temporal resampling, 5000 random pixels were extracted from each individual satellite scene. From these, a random subset of two-thirds were used to generate the training data and the other one-third were retained as independent validation data. This amounted to 7 million pixels for the training dataset and 5 million pixels for the validation dataset. The validation dataset was used for parameter tuning and to evaluate the RF performance on independent data after model fitting. Prior to the model training, the training dataset was further reduced to 5 million random pixels. It was randomly divided into 3 million pixels for model training and the remaining 2 million pixels were used for out-of-bag (OOB) testing.

**Table 1:** Overview of the ML input features.

MSG SEVIRI VIS channels	Satellite angles	Solar angles
MSG SEVIRI VIS006	Azimuth Angle	Zenith Angle
MSG SEVIRI VIS008	Elevation Angle	Declination Angle

#### Hyperparameter Tuning

**Fig. 5:**
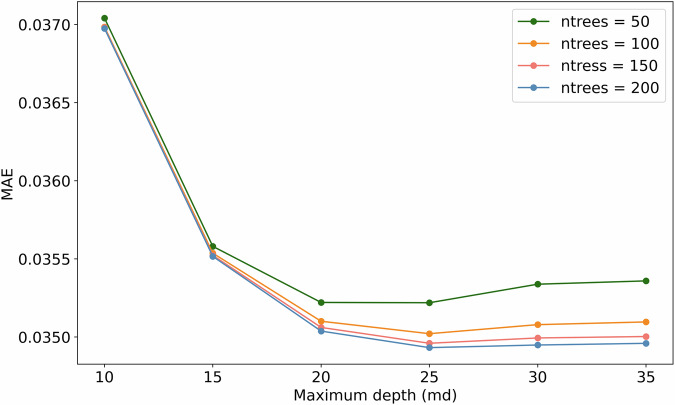
Hyperparameter tuning results for different ntree and md combinations, evaluated using MAE (lower is better).

RF offers the ability to tune the input hyperparameters to optimize the model for performance and efficiency. For this study the RF input hyperparameters (i) number-of-trees (ntrees), (ii) the maximum depth (md), and (iii) random number of predictors per split (mtry) were tuned. md specifies how many levels each decision tree can have, ntrees how many decision trees the model builds, and mtry states how many features the model looks at when making each split in a decision tree. The hyperparameters were tested using an iterative approach with ntrees ranging from 50 to 200 and the md ranging from 10 to 35. The Mean Absolute Error (MAE) was calculated using the validation data set to assess the performance of the RF model for each hyperparameter combination. The results are shown in Fig. 5. The small MAE range of 0.035 to 0.037 for different hyperparameter combinations shows that the performance of the RF model can only be slightly improved by tuning the hyperparameters. The RF model reaches its saturation at 25 md. The ntrees of 200 shows the highest RF model accuracy for all md combinations. Accordingly, md = 30 and ntree = 200 were used as the final hyperparameters for the RF model. It is suggested by^26^ to set the mtry to one third of the model predictors. Since 6 different predictor variables were used, an mtry = 2 is was set accordingly.

#### RF Model Performance

To estimate the performance of the RF model on different subsets of the training data and assess its predictive power, a threefold cross-validation R^2^ score and an out-of-bag (OOB) R^2^ score was calculated^26,31^. Both R^2^ scores measure 0.93 and indicate a strong fit to the MFG MVIRI VIS data (Table 2). The accuracy metrics, mean absolute error (MAE) and root mean squared error (RMSE), were calculated using independent validation data that were not used for model training. For the independent validation data without pixels above 62° latitude or 80° SZA, the final model achieved high prediction accuracy, with an MAE of 0.03/3% and an RMSE of 0.06/6%. The model performance is further discussed in the ‘Technical Validation’ section.

**Table 2:** Performance scores and accuracy metrics of the RF model.

	MAE	RMSE	CV R^2^	OOB R^2^
**Validation data with SZA > 80**°	0.08	0.17	—	—
**Validation data without SZA > 80**°	0.03	0.06	0.93	0.93

#### Feature Importance

**Fig. 6:**
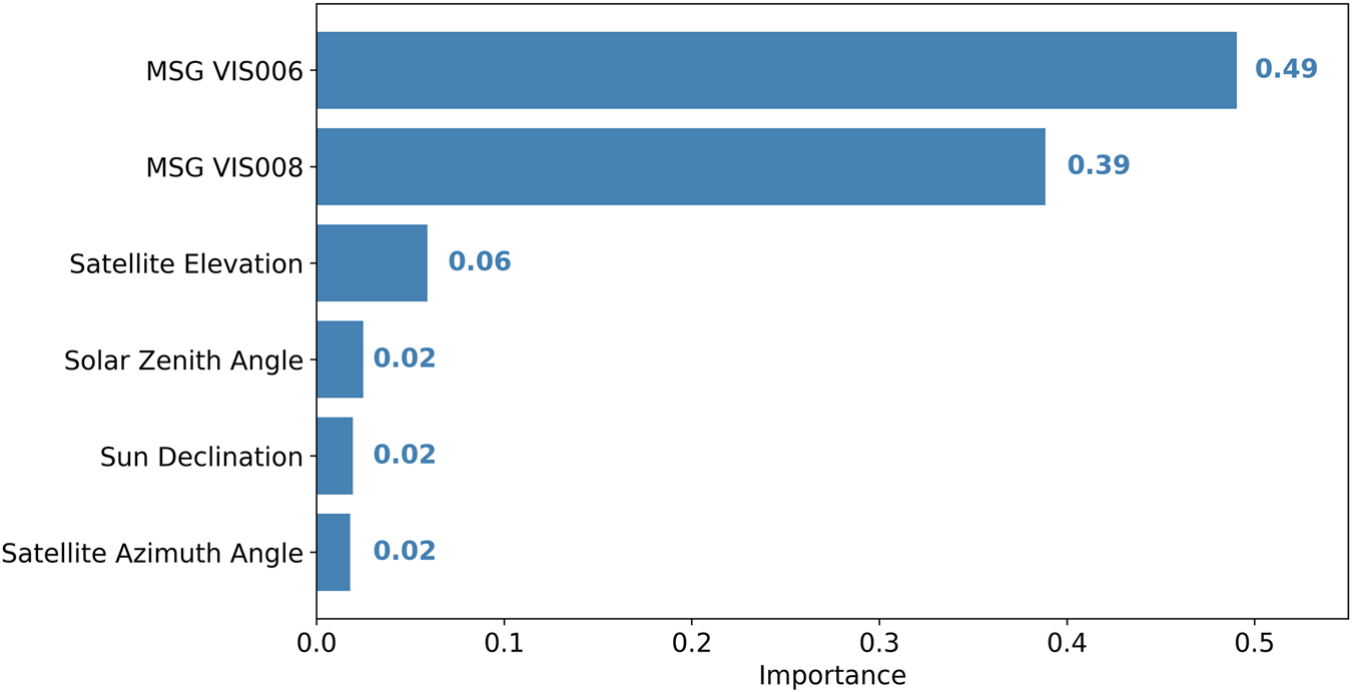
Feature importance of the different RF input predictors.

The feature importance values for each input predictor variable are displayed in Fig. 6. Overall, the two MSG SEVIRI spectral channels have the highest model contribution with 39% - to 49%. The satellite viewing angles and the solar angles have much lower feature importance values of 6% or less. Despite the low feature importance of the satellite and solar angles, tested models without these variables had significantly lower performance scores of more than 10%, suggesting that they are valuable predictor variables.

## Data Records

The synthetic MVIRI Level 1.5 VIS channel Image dataset is available at the data_UMR Research repository^17,32^ under CC-BY 4.0 license. The dataset comprises MFG MVIRI VIS channel images with reflectance values per pixel for the WMO-Region VI Central Europe over the period from July 2006 to December 2020. Each image covers an area from +30.1 to 80.4° latitude and -12.6° to +21.0° longitude. Reflectance values per image were only synthesized for pixels with latitudes below 62°N and with SZA below 80° (see Methods). The spatial resolution is identical to that of the Level 1.5 MFG MVIRI FCDR VIS image data (2.5 km at sub-satellite point). The data are formatted in the Network Common Data Form (netCDF .nc). Images are provided in 30 minute temporal resolution, where two corresponding MSG MVIRI Level 1.5 files were available as input images. The files are stored in folders which are subdivided into year, month and day. Each folder per year is compressed into tape archive GZIP (tar.gz) archives. Each compressed folder is approximately 5 to 7 GB in size. The whole data volume is 100 GB in size.

## Technical Validation

### Model Validation

The RF model was evaluated using two independent validation datasets: one excluding pixels with a SZA greater than 80°, and the other including them. The accuracy metrics are listed in Table 2. The MAE (0.08/8%) and RMSE (0.17/17%) are notably higher for the validation data including dawn and dusk pixels with a higher SZA than for the validation data excluding them (MAE 0.03/3%, RMSE 0.06/6%). The results indicate that the model performs well in predicting the SZA-masked validation data with minor deviations. The higher MAE and RMSE for the non-masked validation data indicates insufficient predictions of reflectance values during dawn and dusk. This further emphasizes the importance of excluding data points during twilight periods with higher SZA values from the model input and training data. Therefore, MFG MVIRI VIS broadband data were only synthesized for pixels with SZA below 80°. This chosen threshold aims to balance the area of applicability and accuracy. Another error source may be introduced by the resampling of the MSG SEVIRI data. Differences in pixel sizes and scan times between the two datasets can result in the mixing and/or misalignment of bright and dynamically developing surfaces, such as clouds, within individual pixels. This leads to reduced spatial accuracy and pixel representation. The validation and homogeneity of the synthetic data set and implications of the average error are further discussed down below and in the Usage Notes section.

**Fig. 7:**
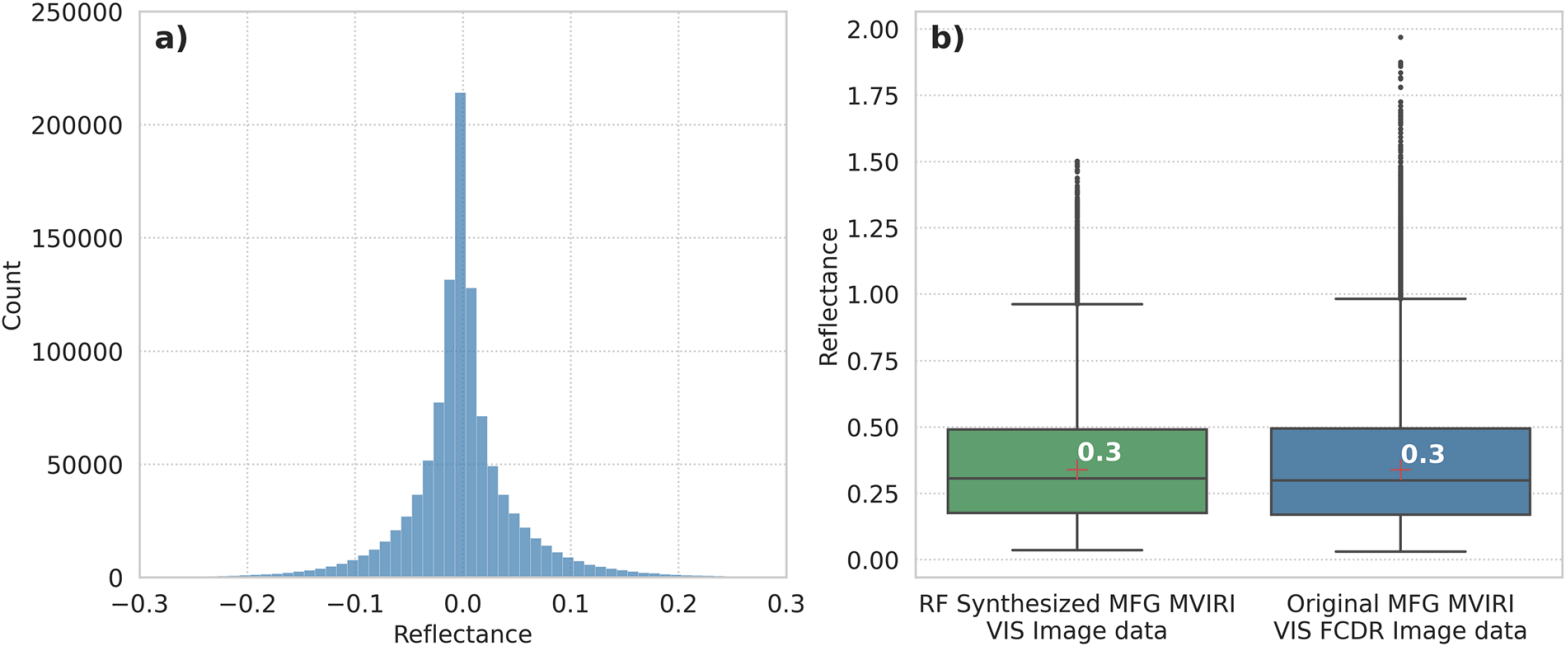
**a**) Histogram of reflectance differences between synthesized and original MFG MVIRI VIS image data. **b**) Boxplot for the original and synthesized values of the MFG solar channel for the overlap period.

To further validate the RF model and estimate its predictive performance, 1000 MFG MVIRI VIS scenes of the overlap period from January 2004 to July 2006 were synthesized using the trained RF model. To ensure the independence of the validation scenes, only MSG SEVIRI scenes that were not used to generate the initial training and validation datasets were used for this process. Figure 7 and Table 3 indicate an overall good fit of the synthesized MFG MVIRI VIS data to the original MFG MVIRI VIS data with only minor deviations. Overall, the RF model tends to under- or overestimate extreme values, which reduces the occurrence of outliers (Fig. 7). This may be due to the RF averaging the output of multiple decision trees, which can ultimately lead to an underestimation of high values and an overestimation of low values^26^. Overall, the average difference between the independent subset of the original and synthesized MFG MVIRI data is well below 1%.

**Table 3:** Statistical metrics of the original and RF-synthesized data MFG MVIRI VIS scenes for the overlap period from 1000 independent scenes.

	25-%tile	75-%tile	mean	sd	median
**Original MFG VIS**	0.169	0.494	0.339	0.212	0.298
**Synthesized MFG VIS**	0.176	0.490	0.339	0.205	0.306

**Fig. 8:**
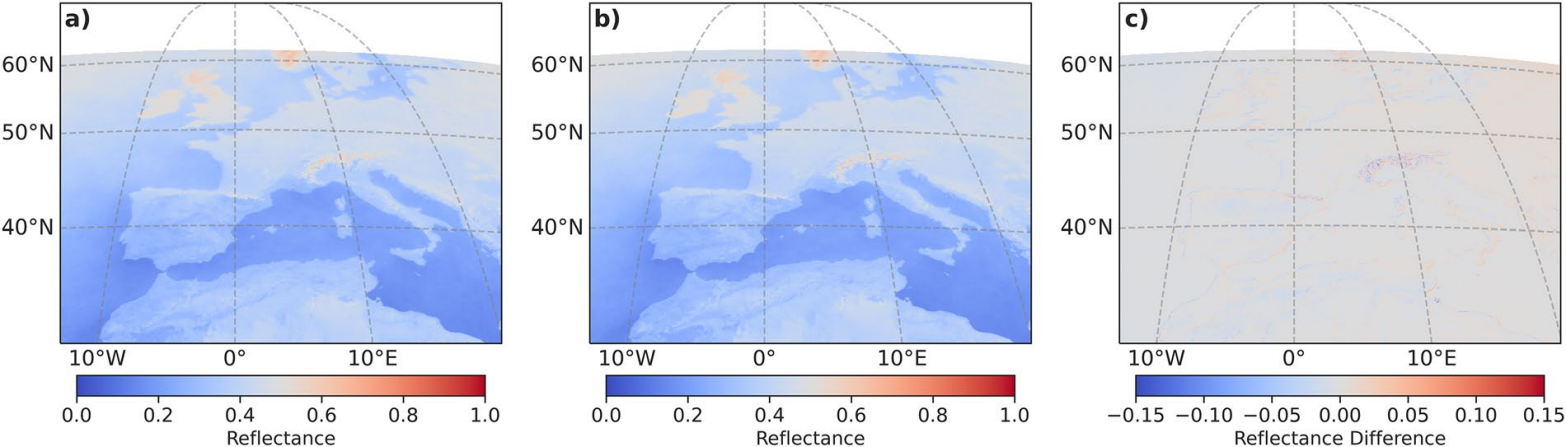
Mean composites and mean difference composites between original MFG and RF-synthesized MFG solar channel reflectance values. **a**) Original scenes, **b**) Synthesized scenes, **c**) Mean difference (original - synthesized).

To test the spatial performance of the RF model, mean composites were generated from the independent scenes (Fig. 8). The RF model shows a high capability to accurately predict the MFG MVIRI VIS data with reflectance differences close to 0 over the entire study area. However, artifacts with noticeable reflectance differences (maximum negative difference = –0.187, maximum positive difference = 0.139) appear in mountainous areas such as the Alps and Pyrenees or along coastlines. This could be caused by a small sample size of training pixels in these areas due to their limited extent or by the nearest neighbor spatial resampling, which can lead to an averaging effect in complex terrain areas with higher variability of reflectance values. Overall, inaccuracies occur in areas with increasingly complex terrain with steep elevation gradients and contrasting surfaces like snow-covered and snow-free areas or land-sea transitions besides each other. Due to the MFG pixel size, pixels can include areas with different terrain or surfaces, like mountains and valleys or snow-free and snow-covered areas, and therefore may not be spatially representative of an area. In addition, mountains can cause poor illumination conditions due to shadowing^30,33^.

**Fig. 9:**
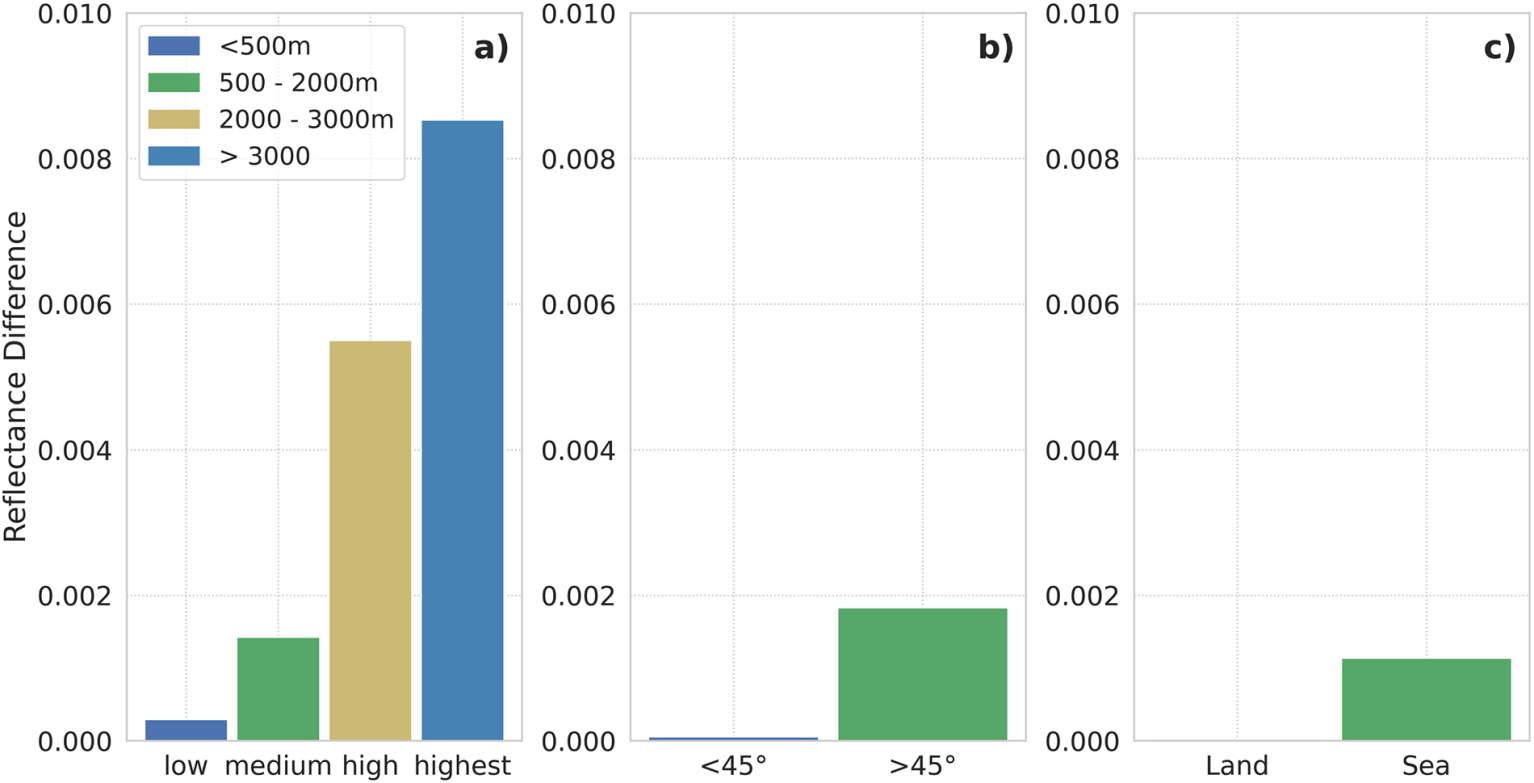
Mean differences of original and RF-predicted MFG MVIRI data for different classes/areas: **a**) Elevation; **b**) Satellite Elevation (0° at the disk edges and 90° at nadir); **c**) Land-Sea Surfaces.

The RF model’s capacity to synthesize the MFG VIS broadband channel data was also evaluated by measuring the mean discrepancy between the synthesized and the original MFG MVIRI VIS channel data for various classes and areas such as (1) elevations; (2) satellite elevation angles; (3) over land or sea surfaces. (Fig. 9). The elevation and land or sea pixel information was extracted from the WorldClim DEM^34^. The elevation information was used to calculate the satellite elevation angle in conjunction with the latitude, longitude and the orbital position (latitude, longitude, altitude) of the Meteosat satellites. As shown in Fig. 9, the RF predictions become less accurate at higher elevations. In general, the average deviation of the RF synthesized MFG VIS data is only marginal with a deviation of less than 0.01/1% for all three classes.

**Fig. 10:**
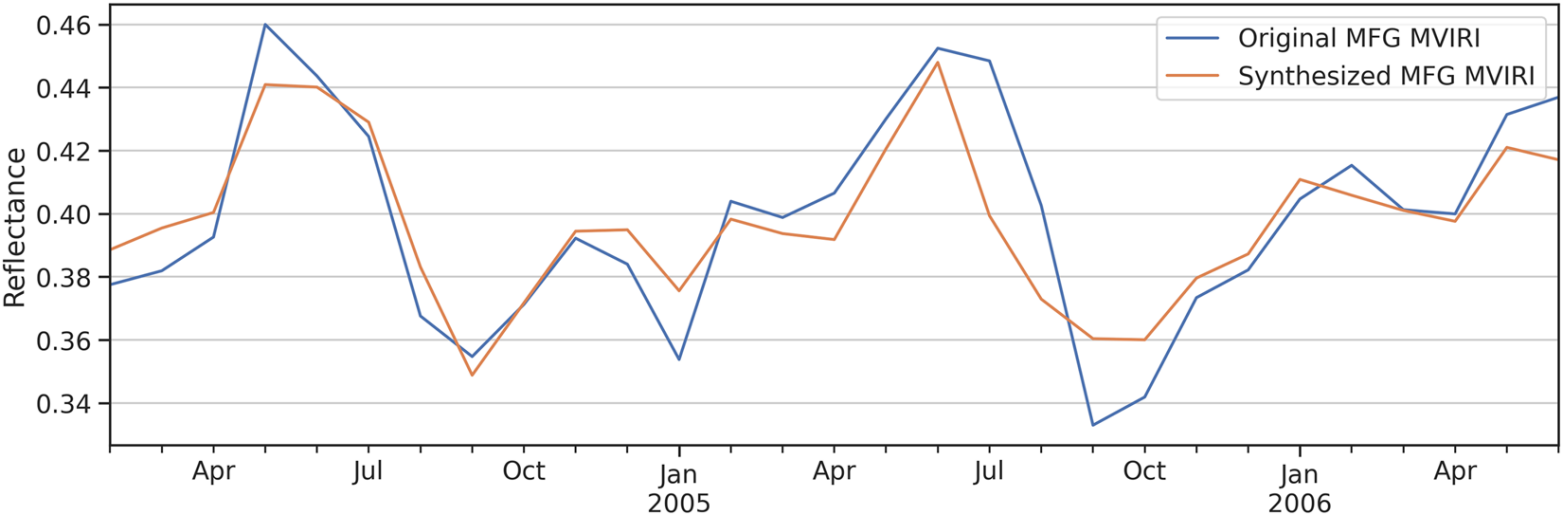
Original and synthesized MFG MVIRI time series of monthly averages for the validation overlap period.

**Fig. 11:**
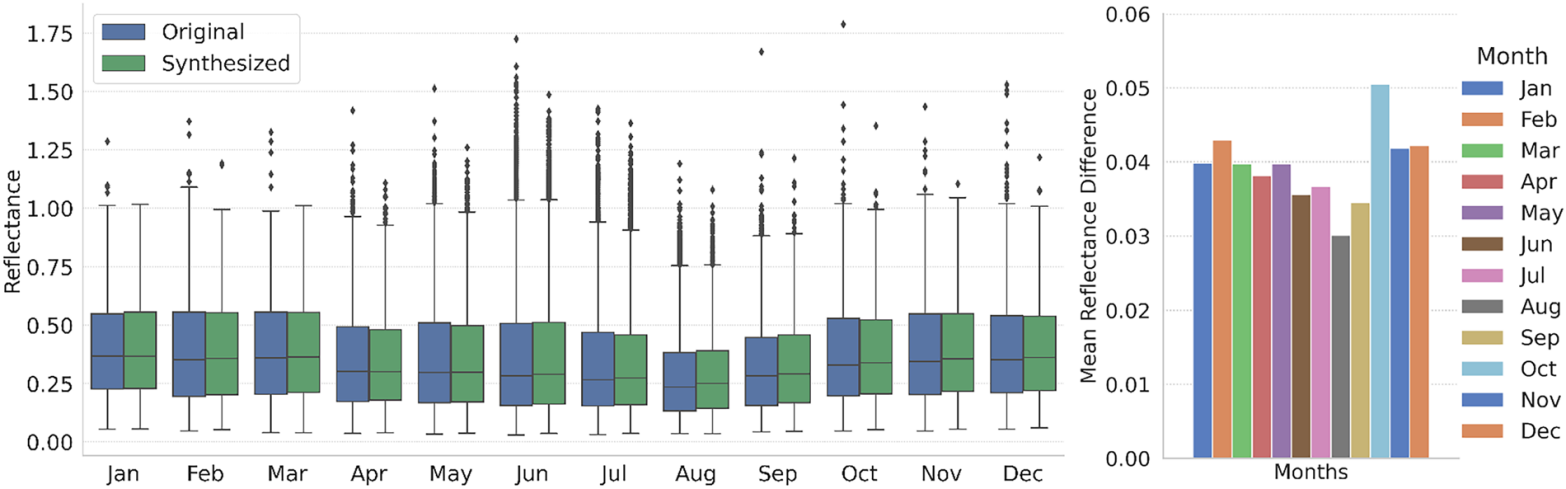
Absolute mean reflectance differences and box plots per month for the original and RF-synthesized MFG MVIRI reflectance data. Box plot whiskers extend 1.5 times of the interquartile range.

The comparison between the monthly averaged original and synthesized MFG MVIRI time series shows that generated data matches the original data well and matches its trend over the validation period (Fig. 10). The average difference between the two data sets is 0.3%. The monthly box plots of the original and the synthesized MFG VIS broadband channel data were created to detect possible inhomogeneities for specific months in the RF predicted data (Fig. 11). The box plots were generated using 1 million random pixels per month from the independent validation scenes. The monthly box plots indicate a good fit of the predicted to the original MFG VIS data. The absolute reflectance differences for each month ranges from 0.03 to 0.05 (Fig. 11). The deviation is slightly higher in the winter months from October to February.

Overall the RF is able to predict the MFG MVIRI data accurately. The average difference between the independent subsets of the original and synthesized MFG MVIRI data is well below 1% despite an MAE of 0.03/3% and an RMSE of 0.06/6%. These are mainly caused by two things: (1) spatial and temporal mismatching of individual pixels of the two data sets due to the differing resolutions and the resampling processes. This appears mainly in alpine areas; (2) Areas with remaining higher SZA close to 80° during twilight periods.

### Data Record Stability and Homogeneity

Ensuring homogeneity and stability of the synthetic MFG MVIRI VIS data is critical to making it a suitable data record for future research. Given the usage of the MSG SEVIRI VIS channel data as RF input data, there is no comparable dataset from 2006 onwards, that can be used as a suitable reference data to validate the synthetic MFG MVIRI Level 1.5 Image data. Since the primary motivation for creating of this dataset was to extend the MFG MVIRI Level 1.5 FCDR VIS Image dataset (Release 1), it is therefore tested for stability and homogeneity compared to the original MFG MVIRI Level 1.5 FCDR VIS image reflectance data. To identify discontinuities in the data related to changes in Meteosat satellites and the switch from original to synthesized MFG MVIRI data (checkpoints, see Table 4), mean reflectance values within one year before and after the checkpoint were calculated. The results are shown in Table 4. The minimal differences, with a maximum difference of 1.7% around checkpoint II, indicate no anomalies in the data around satellite changes. Minor fluctuations in the data are also inherent due to changes in cloud cover and continental surfaces.

**Table 4:** MFG VIS channel mean reflectance values before and after the checkpoints.

Checkpoints	Year	Description	Mean before	Mean after	Difference [%]
**I**	**1994**	**Switch to Meteosat-5**	0.376	0.370	0.6
**II**	**1997**	**Switch to Meteosat-6**	0.382	0.366	1.7
**III**	**1998**	**Switch to Meteosat-7**	0.365	0.368	0.4
**IV**	**2006**	**Switch to synthetic MFG MVIRI**	0.372	0.362	1.1
**V**	**2013**	**Switch to Meteosat-8**	0.361	0.372	1.0
**VI**	**2018**	**Switch to Meteosat-9**	0.356	0.362	0.6

**Fig. 12:**
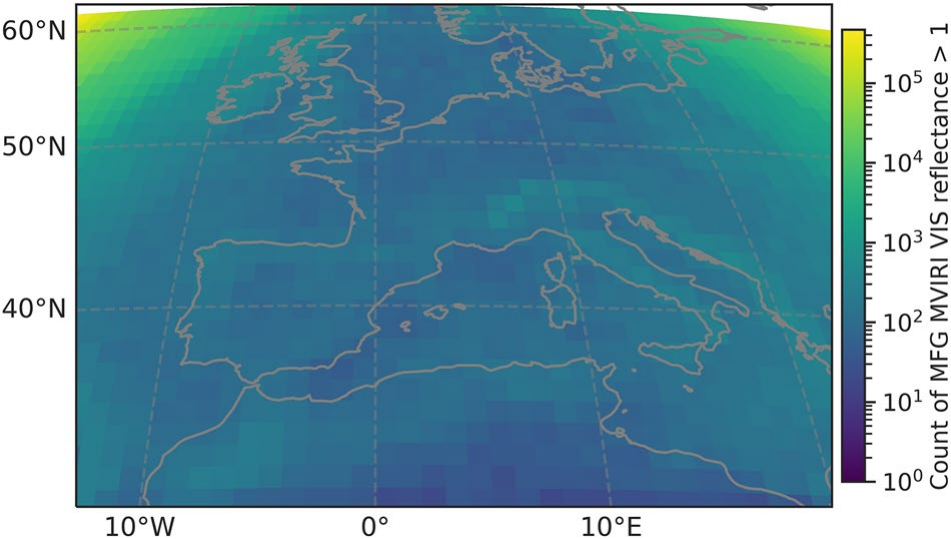
Heatmap of outlier occurrence in the combined MFG MVIRI VIS reflectance data throughout 30 years, 1990 - 2020.

**Fig. 13:**
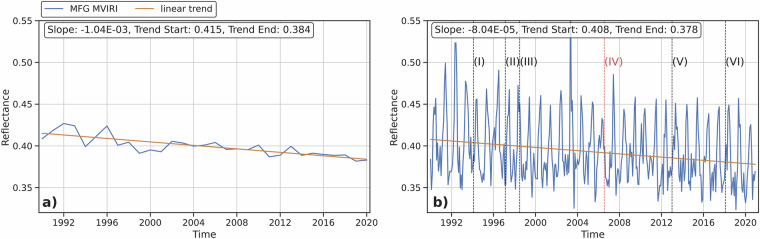
Unfiltered synthesized MFG MVIRI reflectance values, **a**) Annual averaged combined MFG MVIRI VIS channel reflectance data with linear trend, **b**) Monthly averaged combined MFG MVIRI VIS channel reflectance data with linear trend and checkpoints.

Figure 13 shows the monthly averaged (b) and annual averaged (a) MFG MVIRI VIS channel reflectance data from the original MFG MVIRI FCDR and the CDR synthesized by RF over a 30-year period from 1990 to 2020. In general, the combined MFG MVIRI data appear to be stable and homogeneous with a negative trend in the original and synthesized MFG MVIRI VIS channel data during the 30 year period (Fig. 13). It is important to determine whether the trend is caused by the underestimation of outliers and extreme values in the synthesized data, or if it is inherent due to changes in sensor characteristics, operational calibration, or environmental factors such as decreasing cloud cover or changes in continental surfaces. As can be seen in Fig. 12, the outliers occur mainly at the edges of the scenes and are related to pixel distortion and illumination issues in these areas, as described in the Methods section. To further test the stability of the combined MFG MVIRI data, it is necessary to subset the data to valid reflectance values (<1) and remove the outliers. Due to the location of the outliers, this has minimal impact on the quality and applicability of the data (Fig. 12).

**Fig. 14:**
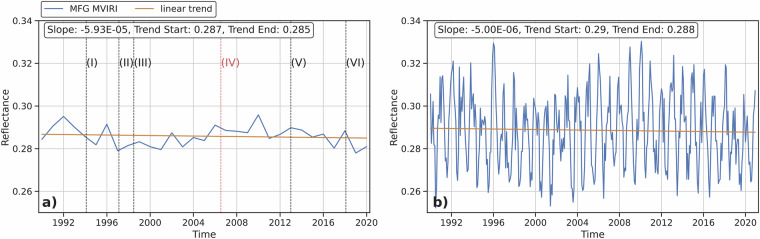
Lower reflectance subset (<0.6), **a**) Annual averaged combined MFG MVIRI VIS channel reflectance data with linear trend, **b**) Monthly averaged combined MFG MVIRI VIS channel reflectance data with linear trend and checkpoints.

**Fig. 15:**
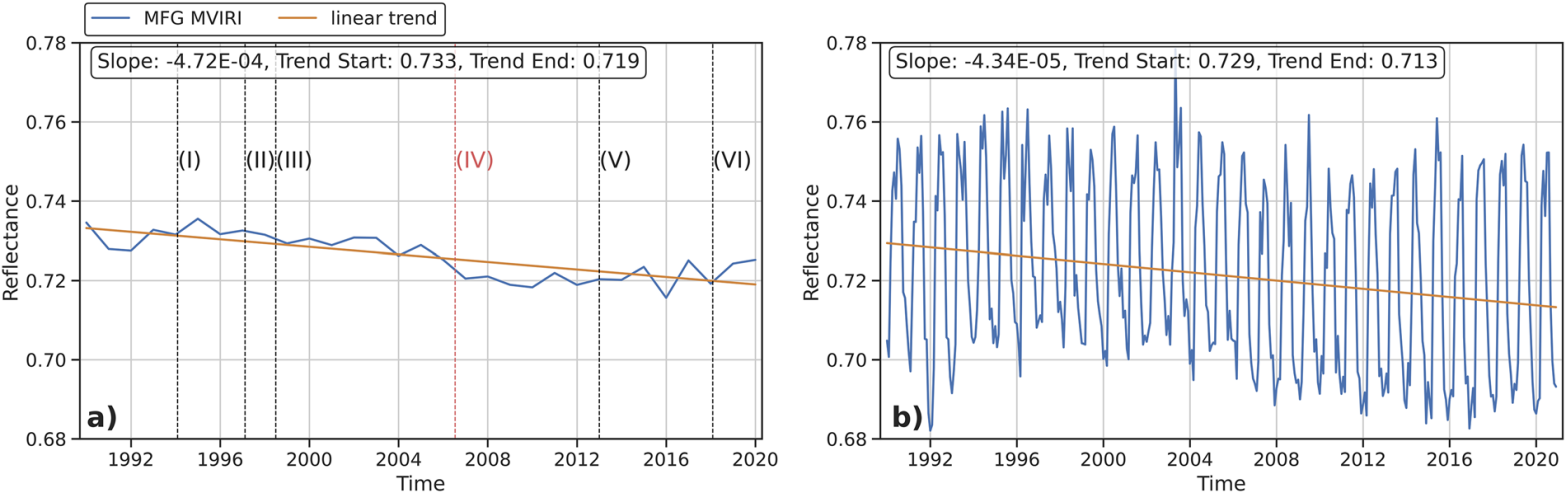
Higher reflectance subset (0.6 - 1), **a**) Annual averaged combined MFG MVIRI VIS channel reflectance data with linear trend, **b**) Monthly averaged combined MFG MVIRI VIS channel reflectance data with linear trend and checkpoints.

To further analyze the performance and stability of the synthesized and/or combined MFG MVIRI VIS channel reflectance data, the combined data was divided into a lower reflectance (0 – 0.6) and a higher reflectance (0.6 – 1) subset. The higher reflectance subset contains mostly cloudy pixels or pixels with icy or snowy surfaces due to their high reflectivity, while the lower reflectance subset captures mostly cloud-free pixels and the remaining surfaces with lower reflectivity (Figs. 14,15). This approach is only a rough estimate, as removing cloudy pixels from remotely sensed data is a complex process. However, due to the lack of a consistent cloud mask product that covers the entire area of interest and time period, a more accurate approach could not be applied. As shown in Fig. 14,15, the observed negative trend is exclusive to the higher reflectance subset with an average decrease in reflectance of 1.4 to 1.6%, which is much lower than the unfiltered synthesized MFG MVIRI VIS channel data. The lower reflectance subset displays a consistent pattern over the 30-year period, with no evident trend. This signifies the stability and homogeneity of the synthesized MFG MVIRI VIS data record. Although the negative trend is minimal, it indicates a decline in high reflectance pixels, which could be associated with the thawing of ice and snow surfaces and a reduction in cloud formation due to global warming^1,3^.

**Fig. 16:**
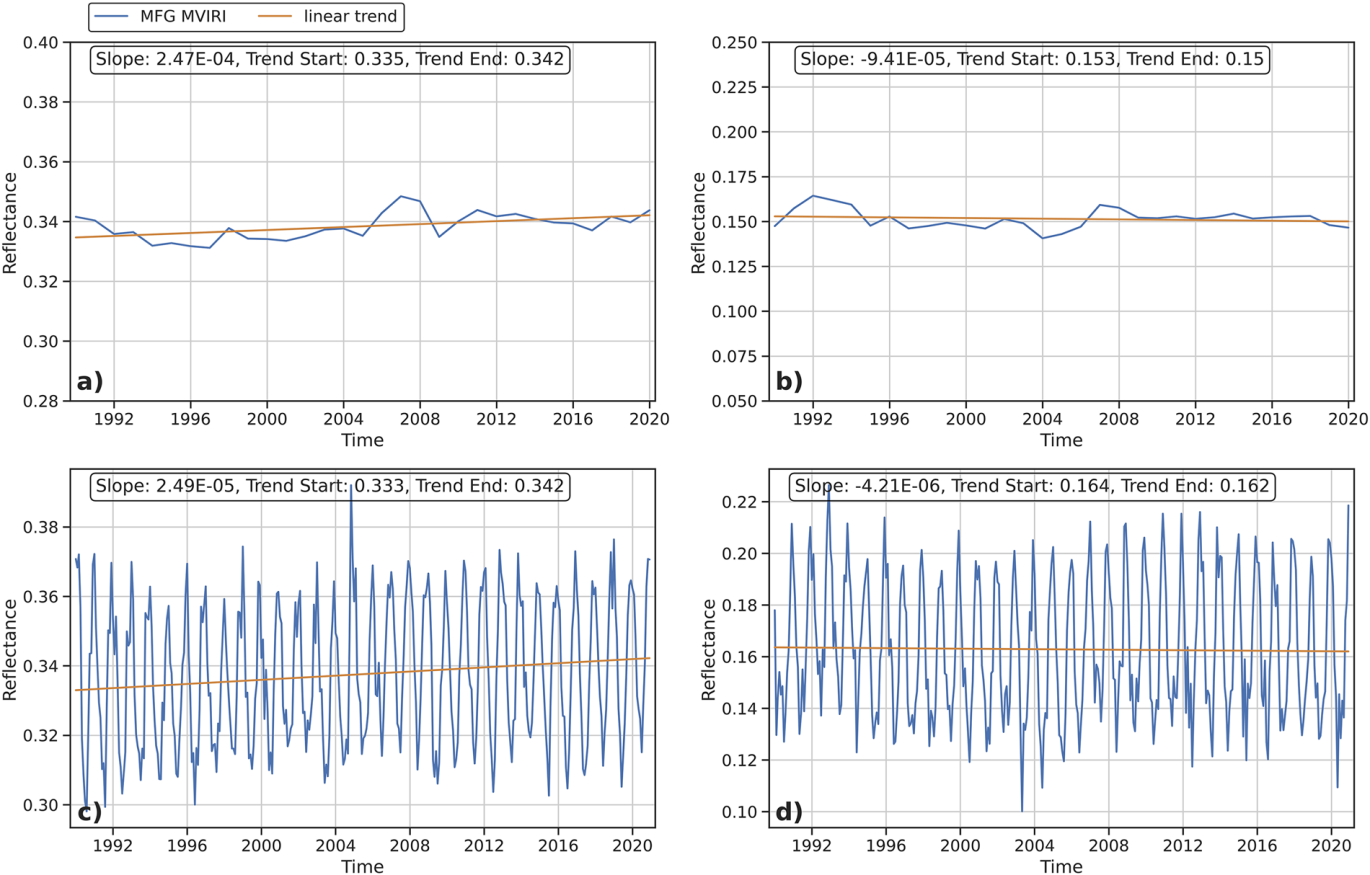
**a**) Annual average reflectance values over clear-sky desert pixels, **b**) Annual average reflectance values over clear-sky ocean pixels, **c**) Monthly averaged reflectance values over clear-sky desert pixels **d**) Monthly averaged reflectance values over clear-sky ocean pixels.

Clear sky ocean and desert pixels were extracted to further confirm the stability of the synthesized and combined MFG MVIRI VIS dataset, as these surfaces naturally have low variability in reflectivity. A similar approach is used to identify suitable calibration pixels for the operational vicarious calibration of the MFG MVIRI and MSG SEVIRI VIS channels^35^. However, small differences are still inherent (around ± 1%), due to naturally changing surface reflection because of waves and sea swelling, algae, different levels of surface moisture and more. For the desert pixels, cloudy pixels and sandstorms were identified by analyzing the daily variation of the observed values^35^. The clear sky pixels were filtered by fitting a second order diurnal polynomial. Any observations that deviate substantially from the polynomial are interpreted as being contaminated by clouds or sandstorms. If a large number of observations per day are filtered, the entire observation for that day is discarded. Cloud-contaminated ocean pixels were filtered using a simple threshold approach, where all observations with reflectance values > 0.3 are discarded. The MFG MVIRI reflectance time series for clear sky ocean and desert pixels are shown in Fig. 16. As can be seen in Fig. 16, there are no pronounced trends or unusual anomalies in the reflectance values for either the ocean or the desert pixel subset, as the mean differences are less than 1% over the 30-year period or between years. This further indicates a highly stable and homogeneous performance of the synthesized MFG MVIRI VIS image data, in addition to the original MFG MVIRI FCDR VIS image data. Additionally, mean values for the original (01-01-1990 to 17-7-2006) and the synthesized (17-07-2006 to 31-12-2020) MFG MVIRI VIS image data were calculated and compared (Table 5). The results show a very stable behavior of the synthesized data compared to the original data after outliers are removed, with very small differences. The highest differences occur in the data without outliers and in the high reflectance subset (0.9%), due to the negative trend associated with possibly decreasing cloud and snow or ice cover. The other subsets show almost no difference (less than 0.6%) between the average of the two data sets. These results also correspond to interannual changes of less than 1% for the entire 30-year time series, as well as for the original and synthesized MFG MVIRI time series individually. Overall, the results demonstrate that the synthesized MFG MVIRI VIS image data have a high stability and homogeneity, thereby indicating their suitability to extend the existing MFG MVIRI FCDR VIS image data.

**Table 5:** Averages of different subsets for the original (1990 – 2006) and the synthesized (2006 – 2020) MFG MVIRI VIS Reflectance data.

Data subsets	Mean original MVIRI	Mean synthesized MVIRI	Difference [%]
**All**	0.407	0.391	1.7
**Outliers removed**	0.370	0.361	0.9
**High reflectance (0.6 - 1)**	0.730	0.721	0.9
**Lower reflectance (<0.6)**	0.286	0.286	0
**Desert Pixels**	0.336	0.341	0.6
**Ocean Pixels**	0.135	0.136	0.1

## Usage Notes

This data can be used like MFG MVIRI or MSG SEVIRI VIS band data. It was created with the purpose to detect long term trends and anomalies in reflectance over large temporal and spatial scales. The data’s suitability for this purpose is indicated by its small MAE of 0.03. It is advised to use the synthetic MFG MVIRI VIS image data for research in mountainous or coastal areas with care due to the slightly higher prediction errors of the RF model. To further decrease the uncertainty in the data usage, invalid reflectance values above one may be removed from the data set before use. Invalid values are mainly located towards the edges of the scenes during twilight periods, thus slightly limiting the area of applicability slightly.

## Data Availability

The python code necessary to reproduce the results is available at LCRS GitLab repository: https://gitlab.uni-marburg.de/fb19/ag-bendix/harmonizing_synthesizing_mfg_vis.

## References

[1] Goessling, H. F., Rackow, T. & Jung, T. Recent global temperature surge intensified by record-low planetary albedo. *Science* **0**, eadq7280, 10.1126/science.adq7280 (2024). 10.1126/science.adq7280 39636934

[2] Calle, A., Casanova, J.L. and Romo, A. Fire detection and monitoring using msg spinning enhanced visible and infrared imager (seviri) data. *Journal of Geophysical Research: Biogeosciences* **111**, 10.1029/2005JG000116 (2006).

[3] Egli, S., Thies, B., Drönner, J., Cermak, J. & Bendix, J. A 10 year fog and low stratus climatology for europe based on meteosat second generation data. *Quarterly Journal of the Royal Meteorological Society* **143**, 10.1002/qj.2941 (2016).

[4] Henken, C. C., Schmeits, M. J., Deneke, H. & Roebeling, R. A. Using msg-seviri cloud physical properties and weather radar observations for the detection of cb/tcu clouds. *Journal of Applied Meteorology and Climatology* **50**, 1587–1600, 10.1175/2011JAMC2601.1 (2011).

[5] Giering, R. *et al*. A novel framework to harmonise satellite data series for climate applications. *Remote Sensing* **11**, 1002, 10.3390/rs11091002 (2019).

[6] Gaurav, S., Egli, S., Thies, B. & Bendix, J. Harmonization of meteosat first and second generation datasets for fog and low stratus studies. *Remote Sensing* **15**, 1774, 10.3390/rs15071774 (2023).

[7] EUMETSAT. Meteosat series. *European Organisation for the Exploitation of Meteorological Satellites*. https://www.eumetsat.int/our-satellites/meteosat-series, (Last accessed: 2025-06-10) (2024).

[8] Govaerts, Y. M., Rüthrich, F., John, V. O. & Quast, R. Climate data records from meteosat first generation part i: Simulation of accurate top-of-atmosphere spectral radiance over pseudo-invariant calibration sites for the retrieval of the in-flight visible spectral response. *Remote Sensing* **10**, 1959, 10.3390/rs10121959 (2018).

[9] Norris, J. R. & Evan, A. T. Empirical removal of artifacts from the isccp and patmos-x satellite cloud records. *Journal of Atmospheric and Oceanic Technology* **32**, 691–702, 10.1175/JTECH-D-14-00058.1 (2015).

[10] Rüthrich, F. *et al*. Climate data records from meteosat first generation part III: Recalibration and uncertainty tracing of the visible channel on meteosat-2–7 using reconstructed, spectrally changing response functions. *Remote Sensing* **11**, 1165, 10.3390/rs11101165 (2019).

[11] John, V. O. *et al*. On the methods for recalibrating geostationary longwave channels using polar orbiting infrared sounders. *Remote Sensing* **11**, 1171, 10.3390/rs11101171 (2019).

[12] Quast, R., Giering, R., Govaerts, Y., Rüthrich, F. & Roebling, R. Climate Data Records from Meteosat First Generation Part II: Retrieval of the In-Flight Visible Spectral Response. *Remote Sensing* **11**, 480, 10.3390/rs11050480 (2019).

[13] Valiente, J. A., Nunez, M., Lopez-Baeza, E. & Moreno, J. F. Narrow-band to broad-band conversion for meteosat-visiible channel and broad-band albedo using both avhrr-1 and-2 channels. *Remote Sensing* **16**, 1147–1166, 10.1080/01431169508954468 (1995).

[14] Deneke, H. M. & Roebeling, R. A. Downscaling of meteosat seviri 0.6 and 0.8 *μ*m channel radiances utilizing the high-resolution visible channel. *Atmospheric Chemistry and Physics* **10**, 9761–9772, 10.5194/acp-10-9761-2010 (2010).

[15] Cros, S., Albuisson, M. & Wald, L. Simulating meteosat-7 broadband radiances using two visible channels of meteosat-8. *Solar Energy* **80**, 361–367, 10.1016/j.solener.2005.01.012 (2006).

[16] Posselt, R., Mueller, R., Trentmann, J., Stockli, R. & & Liniger, M. A. A surface radiation climatology across two meteosat satellite generations. *Remote Sensing of Environment* **142**, 103–110, 10.1016/j.rse.2013.11.007 (2014).

[17] Jung, I., Gaurav, S., & Bendix, J. *data_UMR Research Repository*. 10.17192/fdr/241 (2025).

[18] Rüthrich, F. *et al*. *MVIRI Level 1.5 Climate Data Record Release 1 - MFG - 0 degree**European Organisation for the Exploitation of Meteorological Satellites*. 10.15770/EUM_SEC_CLM_0009 (2020).

[19] EUMETSAT. *High Rate SEVIRI Level 1.5 Image Data - MSG - 0 degree*. *European Organisation for the Exploitation of Meteorological Satellites*. https://user.eumetsat.int/catalogue/EO:EUM:DAT:MSG:HRSEVIRI (2009).

[20] Raspaud, M. *et al*. Pytroll: An open-source, community-driven python framework to process earth observation satellite data. *Bulletin of the American Meteorological Society* **99**, 1329–1336, 10.1175/BAMS-D-17-0277.1 (2018).

[21] Riihelä, A., Carlund, T., Trentmann, J., Müller, R. & Lindfors, A. V. Validation of cm saf surface solar radiation datasets over finland and sweden. *Remote Sensing* **7**, 6663–6682, 10.3390/rs70606663 (2015).

[22] Güls, I. & Bendix, J. Fog detection and fog mapping using low cost meteosat-wefax transmission. *Meteorological Applications* **3**, 179–187, 10.1002/met.5060030208 (1996).

[23] Benas, N. *et al*. Claas-3: the third edition of the cm saf cloud data record based on seviri observations. *Earth System Science Data* **15**, 5153–5170, 10.5194/essd-15-5153-2023 (2023).

[24] Lee, J. R., Chung, C. Y. & Ou, M. L. Fog detection using geostationary satellite data: Temporally continuous algorithm. *Asia-Pacific J Atmos Sc* **47**, 113–112, 10.1007/s13143-011-0002-2 (2011).

[25] EUMETSAT. *Optimal Cloud Analysis Climate Data Record Release 1 - MSG - 0 degree*. *European Organisation for the Exploitation of Meteorological Satellites*. 10.15770/EUM_SEC_CLM_0049 (2022).

[26] Breiman, L. Random forests. *Machine Learning* **45**, 5–32, 10.1023/A:1010933404324 (2001).

[27] Breiman, L., Friedman, J., Stone, C.J. & Olshen, R.A. *Classification and Regression Trees*. (Taylor & Francis 1984), 10.1201/9781315139470.

[28] Hastie, T., Tibshirani, R., & Friedman, J. Random Forests, in *The Elements of Statistical Learning: Data Mining, Inference, and Prediction* 587–604 (Springer New York, New York, NY, 2009). 10.1007/978-0-387-84858-7_15.

[29] Pedregosa, F. *et al*. Scikit-learn: Machine learning in python. *Journal of Machine Learning Research* **12** (2012).

[30] Müller, R. & Pfeifroth, U. Remote sensing of solar surface radiation – a reflection of concepts, applications and input data based on experience with the effective cloud albedo. *Atmospheric Measurement Techniques* **15**, 1537–1561, 10.5194/amt-15-1537-2022 (2022).

[31] Kuhn, M. & Johnson, K. *Applied predictive modeling*, vol. 26. (Springer, 2013). 10.1007/978-1-4614-6849-3.

[32] Jung, I., Gaurav, S., & Bendix, J. *RESPECT data warehouse*. 10.5678/501d-x724 (2025).

[33] Bruno, D., Antoine, Z., Richard, M. & Rolf, P. Verification of cm-saf and meteoswiss satellite based retrievals of surface shortwave irradiance over the alpine region. *International Journal of Remote Sensing* **31**, 4179–4198, 10.1080/01431160903199163 (2010).

[34] Hijmans, R. J., Cameron, S. E., Parra, J. L., Jones, P. G. & Jarvis, A. Very high resolution interpolated climate surfaces for global land areas. *International Journal of Climatology: A Journal of the Royal Meteorological Society* **25**, 1965–1978, 10.1002/joc.1276 (2005).

[35] Govaerts, Y. M., Clerici, M. & Clerbaux, N. Operational calibration of the meteosat radiometer vis band. *Geoscience and Remote Sensing, IEEE Transactions on* **42**, 1900 – 1914, 10.1109/TGRS.2004.831882 (2004).

